# Diversity of aromatic hydroxylating dioxygenase genes in mangrove microbiome and their biogeographic patterns across global sites

**DOI:** 10.1002/mbo3.490

**Published:** 2017-05-23

**Authors:** Sanderson T. P. de Sousa, Lucélia Cabral, Gileno Vieira Lacerda Júnior, Valéria M. Oliveira

**Affiliations:** ^1^ Research Center for Chemistry Biology and Agriculture (CPQBA) University of Campinas (UNICAMP) Paulínia São Paulo Brazil; ^2^ Institute of Biology University of Campinas (UNICAMP) Campinas São Paulo Brazil

**Keywords:** dioxygenases, Gene library, Microbial biogeography, Phylogeny

## Abstract

Aromatic hydrocarbons (AH), such as polycyclic aromatic hydrocarbons, are compounds largely found in nature. Aromatic‐ring‐hydroxylating dioxygenases (ARHD) are proteins involved in AH degradation pathways. We used ARHD functional genes from an oil‐impacted mangrove area and compared their diversity with other sites around the world to understand the ARHD biogeographic distribution patterns. For this, a comprehensive database was established with 166 operational protein families (OPFs) from 1,758 gene sequences obtained from 15 different sites worldwide, of which twelve are already published studies and three are unpublished. Based on a deduced ARHD peptide sequences consensus phylogeny, we examined trends and divergences in the sequence phylogenetic clustering from the different sites. The taxonomic affiliation of the OPF revealed that *Pseudomonas*,* Streptomyces*,* Variovorax*,* Bordetella* and *Rhodococcus* were the five most abundant genera, considering all sites. The functional diversity analysis showed the enzymatic prevalence of benzene 1,2‐dioxygenase, 3‐phenylpropionate dioxygenase and naphthalene 1,2‐dioxygenase, in addition to 10.98% of undefined category ARHDs. The ARHD gene correlation analysis among different sites was essentially important to gain insights on spatial distribution patterns, genetic congruence and ecological coherence of the bacterial groups found. This work revealed the genetic potential from the mangrove sediment for AH biodegradation and a considerable evolutionary proximity among the dioxygenase OPFs found in Antarctica and South America sites, in addition to high level of endemism in each continental region.

## 1 INTRODUCTION

Mangrove is a particular ecosystem located in the intertidal zone of marine coastal environments and estuarine margins. The importance of this ecosystem lies in its great biological productivity, serving as shelter for several species of fish, crustaceans, mollusks, birds, reptiles, mammals, and microorganisms. However, mangroves around the world have suffered due to anthropogenic activities and consequent contamination with heavy metals, pesticides, polychlorinated biphenyls (PCBs), and other industrial pollutants, including oil spill (Sandilyan & Kathiresan, 2014). PAHs are compounds largely found in the environment (e.g., fossil fuels and plants), which are significantly toxic to the organisms, due to its lipophilic, mutagenic and carcinogenic character (Rengarajan et al., 2015). The PHAs are molecules which contain two or more benzene rings, with low solubility in water, high boiling and melting points, in addition to the highly recalcitrant and bioaccumulative properties (Haritash & Kaushik, 2009).

Aromatic‐ring‐hydroxylating dioxygenases (ARHD) are multicomponent proteins responsible for the first step of Polycyclic Aromatic Hydrocarbons (PAHs) degradation (Lu, Zhang, & Fang, 2011). They consist of a catalytic component with a terminal ring‐ hydroxylating portion linked to a ferredoxin domain. The terminal portion consists of an α‐ and a β‐subunits, of which the α‐subunit is the most highly conserved (Jouanneau, Martin, Krivobok, & Willison, 2011). The ARHDs are present in environmental microbial communities and play a crucial role in the aromatic compound degradation (including phenol, naphthalene, phenanthrene, pyrene, benzopyrene, among others), especially in oil‐contaminated sediments or soils (Lu et al., 2011; Mrozik & Piotrowska‐Seget, 2010; Olajire & Essien, 2014; Seo, Keum, & Li, 2009). The contaminated environments exert a strong selective pressure on the microbial community, altering its taxonomic composition and defining its ARHDs gene diversity (Gutierrez et al., 2013; Liang et al., 2011; Tan et al., 2015). The description of the structural and functional diversity of ARHD genes has been important in the establishment of genetic markers for contaminated environments followed by new insights for bioremediation processes (Gutierrez et al., 2013; Liang et al., 2011; Tan et al., 2015).

Molecular methods have been employed in the studies of microbial biogeography to evaluate taxonomic diversity. Microbial taxa are defined by genetic variation of any genomic locus grouped as operational taxonomic units (OTUs) (Koeppel & Wu, 2013). In recent decades, the development of cultivation‐independent techniques associated with next‐generation sequencing (NGS) have stimulated the investigation of the microbial diversity involved in the PAHs degradation (Flocco, Gomes, Mac Cormack, & Smalla, 2009; Gomes et al., 2007; Jurelevicius et al., 2012; Ma et al., 2015; Wu et al., 2014). In addition, biogeography studies has helped to elucidate the evolutionary relationship (governed by selection, drift, dispersal, and mutation processes) of living creatures through genetic proximity between samples from different environments, and thus understand their distribution patterns in space and over time (Hanson, Fuhrman, Horner‐Devine, & Martiny, 2012). Therefore, the microbial diversity can be evaluated under seasonality effect, unusual natural changes or anthropogenic impact in the ecosystems (Lynch & Neufeld, 2015).

The scarcity of studies on evolutionary relationships and distribution patterns of ARHD genes around the world motivated the development of this work. Since there is a reasonable number of published studies that generated amplicons of ARHD genes, we decided to compile all of this information to try to understand the genetic relatedness among these sequences and their geographic distribution across the globe. For this purpose, sediment samples from an oil‐impacted mangrove site was analyzed through the construction and sequencing of one α‐ARHD gene library. The diversity of ARHD genes involved in hydrocarbon biodegradation pathway in the mangrove sediment was determined and compared to ARHD gene sequences derived from other studies, in an attempt to elucidate patterns of biogeographic distribution of such enzymes at a global scale.

## 2 MATERIAL AND METHODS

### 2.1 Sampling

Sediment samples were collected in a mangrove area affected by an oil spill in the 1980s, located in the Bertioga city, São Paulo State (23º53′49′’ S, 46º12′28′’W) (Andreote et al., 2012). Samples were collected perpendicularly in the mangrove transect (approximately 300 m in total), from three sites separated from each other by at least 30 m as described previously (Andreote et al., 2012). Sampling was performed in triplicate using sterile 50 ml‐polypropylene tubes, which were completely filled with sediment to prevent the entry of air, cooled, and transported to the laboratory for further processing.

### 2.2 Community DNA extraction

The metagenomic DNA from 0.3 g of oil‐impacted mangrove sediment was extracted in triplicate with Isolation PowerSoil^®^ kit (Mobio Labs, Inc. Solana Beach, USA), according to the manufacturer's instructions. DNA quantity and quality were determined by agarose gel electrophoresis (1% w/v) and Nanodrop (Thermo Scientific). After the evaluation, DNA samples were stored at −20°C for subsequent analyzes.

### 2.3 PCR amplification of the ARHD genes

The mangrove metagenomic DNA was used as template for ARHD gene PCR‐based amplification using the degenerate primers: ARHDf (TTY RYI TGY AII TAY CAY GGI TGG G) and ARHDr (AAI TKY TCI GCI GSI RMY TTC CA) (Bellicanta, 2005). These primers were designed to flank a highly conserved region of the alpha subunit from ARHD gene, with an expected amplicon size ranging between 300 and 329 bp. The PCR reaction was prepared to a final volume of 50 μl containing 1X *Taq* buffer (Invitrogen), 1.5 mmol/L MgCl_2_, 0.2 mmol/L dNTP mix (GE Healthcare), 1.2 μmol/L of each primer, 1 U Platinum *Taq* DNA polymerase (Invitrogen) and 2 μl (12 ng/μl) of eDNA. The reaction was conducted in an Eppendorf Mastercycler Gradient (Eppendorf Scientific, New York, USA) and the program consisted of an initial denaturation at 97°C for 3 min, followed by 35 cycles of denaturation at 94°C for 1 min, annealing at 57°C for 1 min and extension at 72°C for 1 min, and a final extension step at 72°C for 5 min. PCR products were separated on 1% agarose gel (Fisher Scientific, MA) in 1X TAE buffer, using the molecular weight marker 1 Kb Plus DNA Ladder (Invitrogen) for size estimation and observed under UV light using a ImageQuant LAS 4000 system (GE Healthcare Life Sciences).

### 2.4 Construction of the ARHD gene library

PCR products were excised from the gel and subsequently purified using Illustra GFX PCR DNA Purification Kit (GE Healthcare Life Sciences). The amplicons were inserted into the cloning vector pTZ57R/T and transformed into *Escherichia coli* JM 109 cells using InsTAclone PCR Cloning Kit (Thermo Scientific), according to the manufacturer's protocols. Transformed cells were plated onto LB agar containing 50 μg/ml of ampicillin, 40 μl of 5‐bromo‐4‐chloro‐3‐indolyl‐β‐D‐galactopyranoside [X‐Gal (20 mg/ml)] and 40 μl of isopropyl β‐D‐thiogalactopiranoside [IPTG (100 mmol/L)], and incubated at 37°C for 18 hr. Positive (white) colonies were transferred to a new LB agar plate containing the same concentrations of ampicillin, X‐Gal and IPTG, and incubated for 20 hr at 37°C in order to confirm the success of the ligation/transformation procedure. White colonies were transferred to 96‐deep well plates with 1 ml of LB broth medium, following incubation at 37°C for 18 hr. After that, aliquots of cell growth were transferred to 96 well‐ELISA plate and added of 10% glycerol for storage at −80°C.

### 2.5 Sequencing of the ARHD gene library

PCR amplification of the ARHD genes from the plasmid clones was performed in 96‐well PCR plates in a final volume of 25 μl using the primers M13F (5′ CGC CAG GGT TTT CCC AGT CAC GAC 3′) and M13R (5′ TTT CAC ACA GGA AAC AGC TAT GAC 3′). PCR reaction contained 1X *Taq* buffer (Invitrogen), 1.5 mmol/L MgCl_2_, 0.2 mmol/L dNTP mix (GE Healthcare), 0.4 μmol/L of each primer, 2 U Platinum *Taq* DNA polymerase (Invitrogen) and 1 μl (10 ng/μl) of plasmid DNA. The reaction was conducted in an Eppendorf Mastercycler Gradient (Eppendorf Scientific, New York, USA) and the amplification program consisted of an initial denaturation at 94°C for 3 min, followed by 30 cycles of denaturation at 94°C for 30 s, annealing at 60°C for 20 s and extension at 72°C for 90 s, and a final extension step at 72°C for 5 min. The PCR products were purified using the Illustra GFX 96 PCR Purification Kit (GE Healthcare Life Sciences) and checked on 1% agarose gel electrophoresis (Fisher Scientific, MA). The purified PCR products were then used as template for sequencing reaction using Big Dye kit (Life Technologies) and the primers M13R, according to manufacturer's guidelines. The sequencing of ARHD gene library was performed using the ABI 3500 XL platform (Applied Biosystems^®^ 3500 XL).

### 2.6 Bioinformatic analysis of ARHD gene sequences

ARHD gene sequences obtained were up‐loaded in Phred software (Ewing & Green, 1998), for the evaluation of the quality of the base calls and sequences with score <20 were removed. The filtered sequences were edited using the Bioedit software (Hall, 1999) to remove the remaining primer and vector sequences. After treatment, the sequences were classified using Blastx tool (Altschul et al., 1990) from National Center for Biotechnology Information (NCBI) against “Reference Protein” database (Refseq_protein). The best hits (returned sequence with best identity) were considered as reference for taxonomic classification.

### 2.7 Phylogenetic analysis

Phylogenetic analyses were performed using the following sequence datasets: (1) ARHD gene sequences generated in this study, and (2) Homologous sequences of public domain obtained from other environments around the world. The following public sequence datasets were used: Pearl River in China (Wu et al., 2014); oil reservoir in Brazil (Silva, Verde, Santos Neto, & Oliveira, 2013); rhizosphere and bulk soil samples from Antarctica (Flocco et al., 2009) Antarctic ice (Kuhn & Pellizari, unpublished); microbial mats from France (Bordenave et al., 2008); coastal marine sediments of Patagonia (Lozada et al., 2008); biphenyl‐contaminated river sediment from United States (Sul et al., 2009); anthropogenic dark earth from Brazilian Amazon (Germano et al., 2012); Pine root zone from Czech Republic (Leigh et al., 2007); soils from King George Bay, Antarctic (Jurelevicius et al., 2012); PAH‐polluted soil from France (Cébron et al., 2011); and soils and sediments from Antarctica (Muangchinda et al., 2015). The main goal of this step was to conduct a biogeographical analysis using a conserved region of the ARHD gene as marker. Sequence alignment was performed using the ClustalW tool (Larkin et al., 2007) in Bioedit v.7.2.5. The aligned sequences were analyzed using Mothur v.1.33.3 (Schloss et al., 2009) to determine the OPFs (Operational Protein Families) at the evolutionary distances (D) 0.03, 0.05, 0.10 and 0.20. The EMBOSS Transeq software was used to convert the OPF DNA sequences to amino acid sequences in the correct reading frame. The best OPF representatives (D = 0.05) were submitted in MEGA v5.0 program (Tamura, Dudley, Nei, & Kumar, 2007) for the construction of the phylogenetic trees. The phylogenetic trees were constructed using Neighbor‐joining method and Kimura 2‐parameter substitution model (Kimura, 1980), with bootstrap values calculated from 1,000 replicate runs.

### 2.8 Statistical analysis

ARHDs richness and diversity were analyzed in Mothur v.1.33.3 and were calculated based on the sequence datasets using Shannon and Simpson diversity indexes, and Chao1 and ACE richness estimators. In addition, rarefaction curves were constructed to assess the observed richness (D = 0.05). The distribution of ARHD OPFs (D = 0.05), considering the sequences from all sites and based on geographic origin, was compared and displayed in a Venn diagram (Fauth et al., 1996). Principal Coordinates Analysis (PCoA) of Bray–Curtis distances was conducted in Past3 software version 3.14, and assessed the genetic correlation among the sites.

## 3 RESULTS

### 3.1 A brief description of the analyzed sites

**Figure 1:**
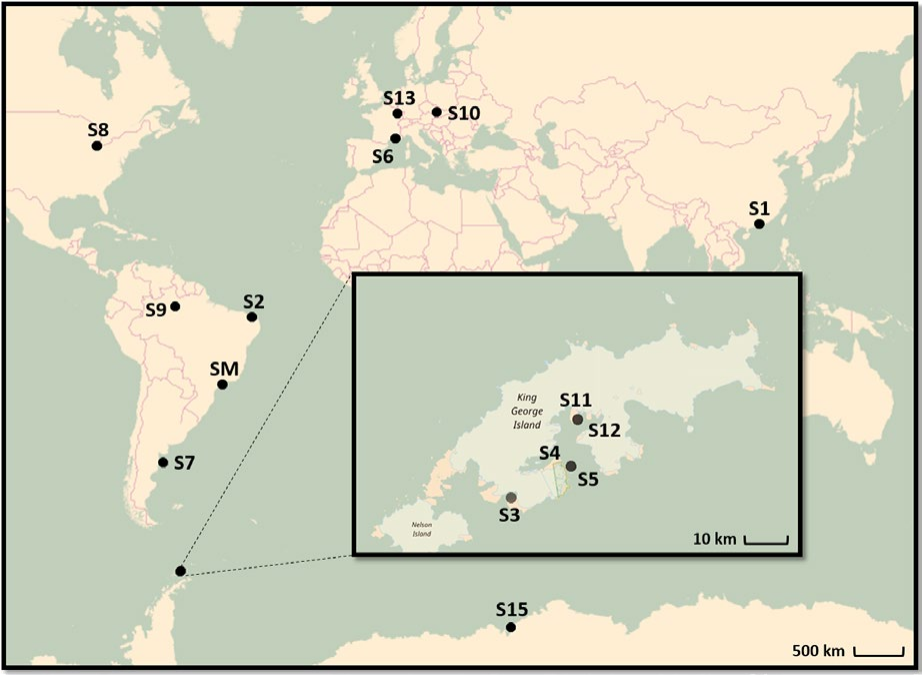
Sampling sites around the world

The biogeographic distribution patterns of the ARHD genes worldwide were obtained through the combined analysis of twelve datasets resulting from already published studies and two unpublished, in addition to the dataset from this study. Of all studies, one was carried out in Asia (River estuary, China), one in North America (River sediment, USA), three in South America (petroleum reservoirs, Brazil; coastal marine sediments, Argentina; dark earth of Amazonia, Brazil), three in Europe (lagoon, south‐east France; pine root zone, Czech Republic; PAH‐contaminated soil, France) and six in Antarctica (Figure 1) (Table 1). All the sequences derived from these studies are available for public access in the Genbank database and were downloaded to proceed with the bioinformatics analyses performed in this study.

**Table 1:** Worldwide sites analyzed in this study

Code	Sites	Reference
SM	Mangrove Sediment, Bertioga city, Brazil	This study
S1	Sediments from the Pearl River estuary, China	Wu et al. (2014)
S2	Oil samples (naturally mixed with formation water) of Potiguar Basin (Northeast, Brazil)	Lima Verde et al. 2013
S3	Soil from Jubany station, King George island, Antarctic	Flocco et al. (2009)
S4	Botany Point, King George island, Antarctica	unpublished
S5	Brazilian station, King George island, Antarctic	unpublished
S6	Pristine and oil contaminated microbial mats, locations in south‐east France	Bordenave et al. (2008)
S7	Coastal marine sediments of Patagonia	Lozada et al. (2008)
S8	Polychlorinated Biphenyl‐Contaminated River Raisin Sediment, river in southeastern Michigan, United States	Sul et al. (2009)
S9	Anthropogenic dark earth of Amazonia	Germano et al. (2012)
S10	Pine root zone contaminated with polychlorinated biphenyls (PCBs), Czech Republic	Leigh et al. (2007)
S11	Different soils from King George Bay, Antarctic Peninsula (sA_sB_sC) ‐ Diesel oil‐contaminated soils	Jurelevicius et al. (2012)
S12	Different soils from King George Bay, Antarctic Peninsula (rookery, Ipanema, yellow soil) ‐ Pristine soils	Jurelevicius et al. (2012)
S13	PAH‐polluted soil, Neuves‐Maisons, France	Cébron et al. 2011
S15	Antarctic soils and sediments around Syowa Station, East Ongul Island, Antarctica	Muangchinda et al. 2015

### 3.2 Description of ARHD gene library

**Figure 2:**
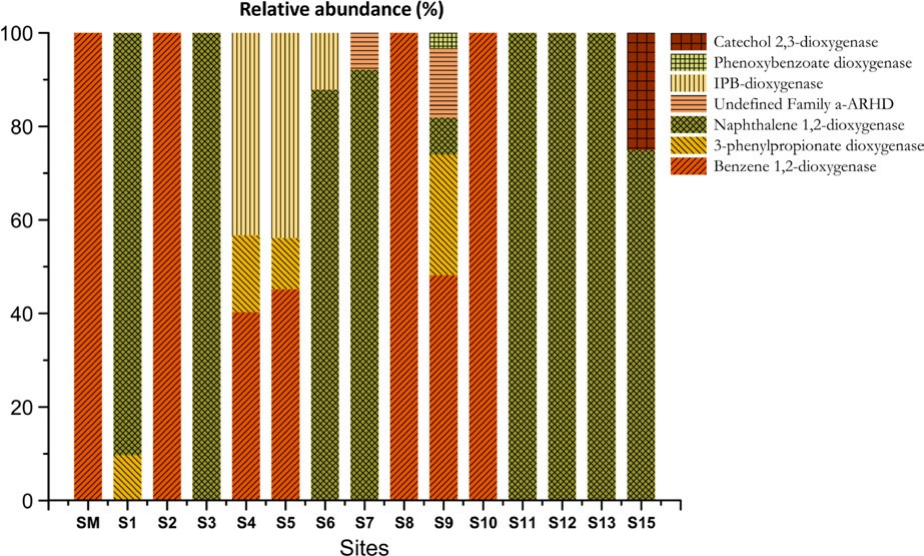
Relative abundance of ARHD sequences in each site based on the functional classification against the NCBI RefSeq database by BLAST

The gene library assembled in this study yielded 129 high quality inserts for ARHD genes (approximately 329 bp in length) from the 288 sequenced clones (44.8%). The functional classification was confirmed by BLASTx and revealed that all sequences were more closely related with the benzene 1,2‐dioxygenase enzyme retrieved from *Pseudomonas* (Figure 2). The average identity between the sequences was 77%, with values ranging from 45% to 94%. Therefore, the majority of OPFs represent new types of ARHD gene, emphasizing their singularity and ecological importance to the mangrove. The search for functional domains revealed the presence of the dioxygenase alpha subunit containing the “Rieske” conserved domain in all sequences of interest.

### 3.3 Phylogenetic analysis of ARHD genes

**Figure 3:**
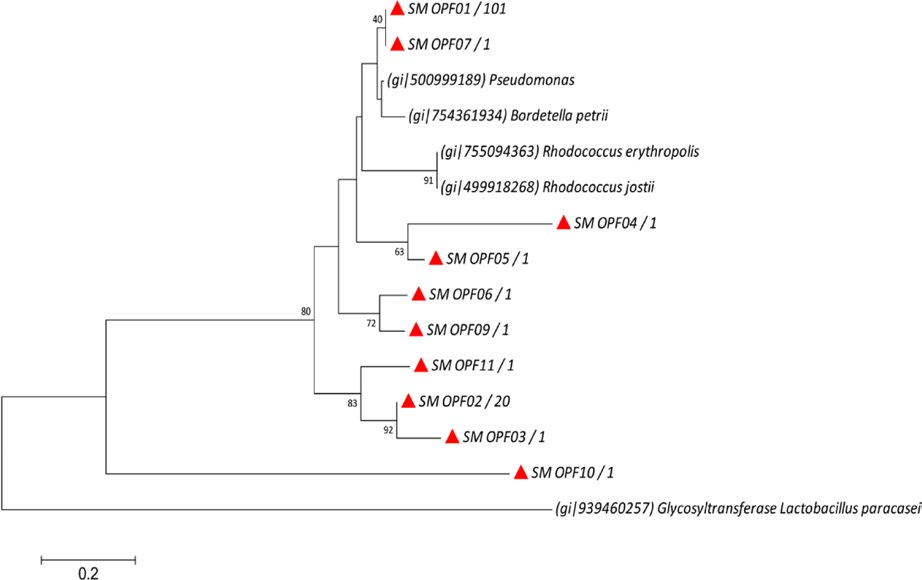
Phylogenetic tree representing the relationships among the OPF representatives for ARHD genes recovered from mangrove sediment and the most related hits from the GenBank database. Bootstrap values were obtained from 1,000 replicates and only values above 40% are shown. The red triangle represents the observed OTUs. The number of sequences in each OPF is shown after the OPF name. The gray scale bar represents 20% sequence divergence. The sequence of the glycosyltransferase from Lactobacillus paracasei was used as an outgroup

Eleven OPFs were identified among the 129 gene sequences determined in this study, considering 0.05 dissimilarity level as cutoff. The deduced ARHD peptide sequences were clustered into five distinct phylogenetic groups (Figure 3). This phylogenetic arrangement showed an unknown catabolic diversity in the mangrove sediment represented by new putative gene sequences. The OPFs 01 e 02 were the most abundant ones, accounting for 78% and 15% of all analyzed clones, respectively. These two OPFs were grouped in different clusters, the OPF01 presented 89% sequence similarity with a benzene 1,2‐dioxygenase of *Pseudomonas*, while the OPF02 showed 78% sequence similarity with the same peptide sequence. The remaining OPFs (03, 04, 05, 06, 07, 09, 10, and 11) were represented by one single clone, accounting for 7% of the total sequences. OPF07 showed a greater relatedness to the OPF01, and they were both recovered into a monophyletic cluster containing two reference sequences obtained from Genbank, one from *Pseudomonas* sp. and other from *Bordetella petrii*. OPFs 02, 03, 04, 05, 06, 09, 10, and 11 formed distinct clusters more distantly related to the reference sequences, with special emphasis to the OPF10, which was recovered in a completely separate branch distantly related to all other groups.

### 3.4 Biogeographic patterns of ARHD genes worldwide

**Figure 4:**
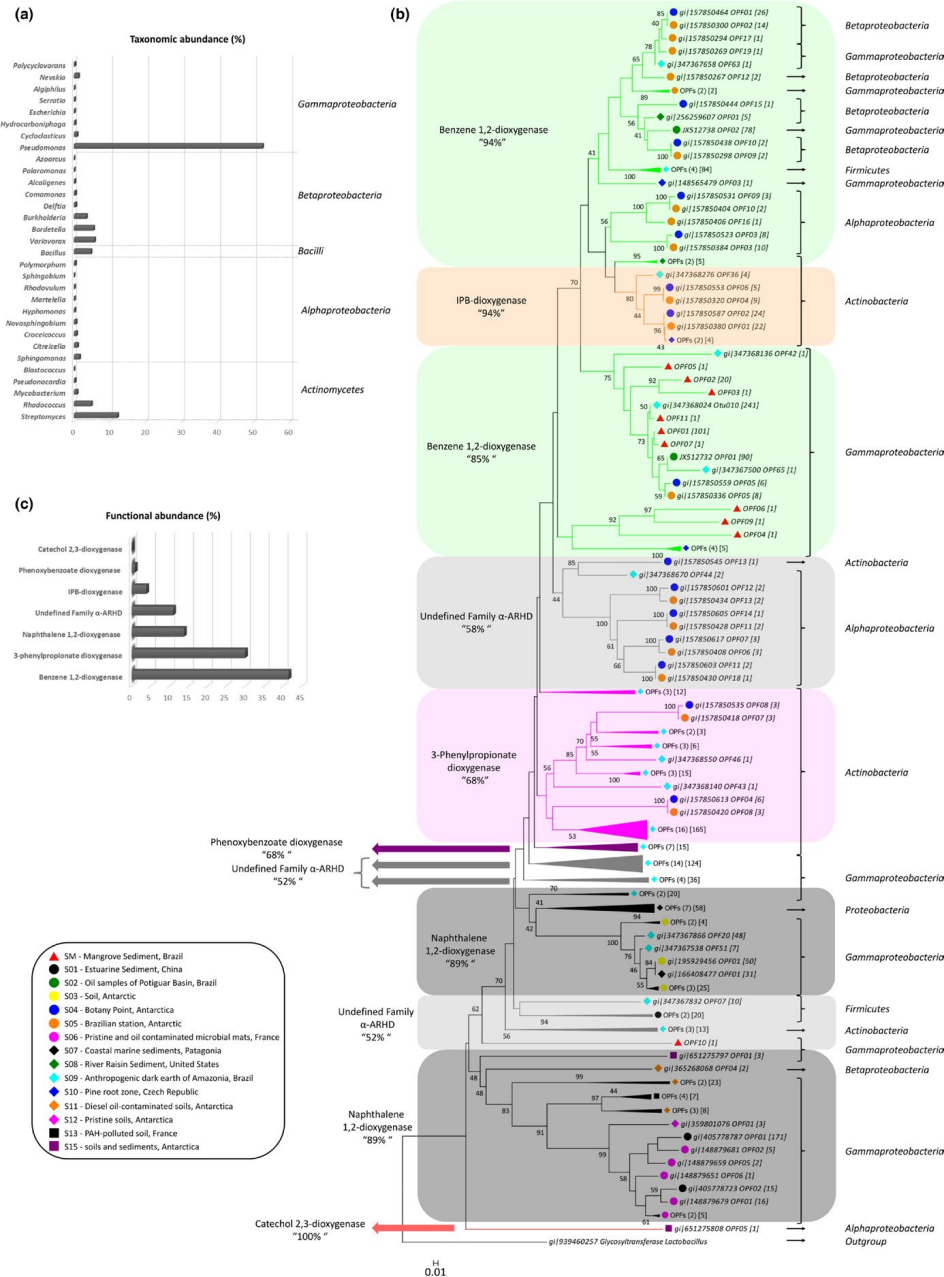
Taxonomic (a) and functional (c) abundance of the total ARHD gene sequences of all sites under study. Phylogenetic analysis (b) of ARHD sequences representing 166 OPF among 1758 ARHD sequences from 15 sites around the world. Bootstrap values were derived from 1,000 replicates and black scale bar represents 1% sequence divergence. Background colors represents the functional categories of ARHD gene sequences based on the classification against GenBank database. The values in double quotes represent the averages of similarity of the OPFs with their closest hits, from the GenBank database, in each functional group of the tree. Total numbers of OPFs are shown in parentheses. Total numbers of gene sequences are shown in brackets. The sequence of the glycosyltransferase from Lactobacillus paracasei was used as an outgroup

Sequence analysis in the Mothur software allowed the identification of the OPF representatives for each site analyzed, totalizing 166 OPFs from 1,758 gene sequences (Table S1). The taxonomic assignment of all OPFs found, considering the five most abundant genera, revealed that 52% of all sequences matched to the genus *Pseudomonas*, 12% to *Streptomyces*, 5.7% to *Variovorax,* 5.4% to *Bordetella* and 4.8% to *Rhodococcus* (Figure 4a). The taxonomic assignment and relative abundance of all sequences analyzed are detailed in Supplementary Table 1. Functional annotation of OPF sequences against the NCBI RefSeq database revealed seven distinct functions. The biogeographic phylogenetic tree was divided into eleven groups considering the OPFs arrangement and their gene function (Figure 4b). Relative abundances were considerably different among the distinct functions, as follows: 41.47% of all the sequences matched to benzene 1,2‐dioxygenase, 29.95% to 3‐phenylpropionate dioxygenase, 13.82% to naphthalene 1,2‐dioxygenase, 10.98% to an undefined ARHD family, 3.87% to IPB‐dioxygenase, 0.85% to phenoxybenzoate dioxygenase and 0.06% to catechol 2,3‐dioxygenase (Figure 4c). The sequences assigned to the benzene 1,2‐dioxygenase were distributed in the sites SM, S2, S4, S5, S8, S9, and S10, representing all continents except Asia (Figure 2 and 4b). Despite being the second most abundant function in general, 3‐phenylpropionate dioxygenase was distributed only in sites S1, S4, S5, and S9, representing Asia, Antarctica and South America (Figure 2). The sequences assigned to the naphthalene 1,2‐dioxygenase were distributed in more than half of the analyzed sites, representing the Asia (S1), South America (S7 and S9) Antarctica (S3, S11, S12, and S15) and Europe (S6 and S13) (Figure 2). The sequences related to the undefined ARHD Family were distributed in two sites in South America (S7 and S9), while the sequences assigned to the IPB‐dioxygenase were distributed in sites located in Antarctica (S4 and S5) and Europe (S6) (Figure 2). Finally, the sequences corresponding to the phenoxybenzoate dioxygenase and catechol 2,3‐dioxygenase were distributed only in one site in South America (S9) and Antarctica (S15), respectively (Figure 2). The sites S4, S5 (Antarctica) and S9 (South America) showed greater functional diversity (Figure 2).

The majority of OPFs in each site (S4, S5, S9, S3, S7, S11, S12, S1 e SM) were clustered closely together, suggesting an overall level of endemism among the distinct OPFs (Figure 4b). The OPFs 05, 02, 03, 11, 01 e 07 determined in this study were grouped with OPFs of the sites S9 (Amazonia, OPFs 42, 10 and 65), S2 (Northeast, Brazil, OPF 01), S4 (Botany Point, Antarctica OPF05) and S5 (Brazilian station, Antarctica, OPF05). The phylogenetic tree showed closer evolutionary relatedness among the OPFs of the sites S4, S5, SM, S2, S9, and S8, representing Antarctica, South America and North America (Figure 4b). Curiously, the OPFs of the site S10 (Europe) also showed evolutionary relatedness to the OPFs of Antarctica and South America sites (Figure 4b).

Of the five OPFs identified among the eight sequences recovered from the S15 site (Antarctic soils), only two were annotated as putative dioxygenases. The OPF05 showed 100% of sequence similarity to a catechol 2,3‐dioxygenase obtained from *Sphingobium yanoikuyae*. Some OPFs of the sites S4 (Antarctica, OPFs 13, 12, 14, 07, and 11), S5 (Antarctica, OPFs 13, 11, 06, and 18) and S9 (Amazonia, OPF 44) were recovered in a tight cluster, except for OPFs 44 and 13, and classified as “undefined ARHD family” because of the low average percentage of identity (<60%) with their closest bacterial hits (Figure 4b). Similarly, a layer of the tree with eighteen OPFs (52% average identity) from the site S9 (Amazonia) and other (52% average identity) containing 4, 2 and 1 OPFs from the sites S9 (Amazonia), S1 (China) and SM (Mangrove sediment), respectively, were classified as “undefined ARHD family” and clustered separately from the other ARHDs (Figure 4b). These OPFs encompassed a large number of sequences, apparently endemic to the Amazonia site.

### 3.5 ARHD gene diversity worldwide

Genetic diversity of the ARHD genes was analyzed at 0.02 and 0.05 distance (D) cutoff (Table 2). However, based on the clusters observed from an initial phylogenetic tree performed with all nucleotide sequences and BLASTP analysis to identify similarities, the cutoff value of 0.05 distance for OPF definition was chosen for subsequent analyses. At 5% distance, 93–100% coverage values were observed for the ARHD gene diversity in all sites examined, except for sites S10 (Czech Republic) and S13 (France), which showed coverage lower than 60% (Table 2). Shannon index calculations showed that ARHD diversity in the mangrove soil under study was relatively higher than in the sites S1 (China), S2 (Northeast, Brazil) and S12 (Antarctica), although lower than the other sites (Table 2). The Simpson index measures the probability that two randomly sorted sequences within a sample will be of the same species (Simpson, 1949). The observed value for the Simpson index (62%) also demonstrated that the mangrove sediment (SM) has a relatively higher diversity (lower probability) than the sites S1, S2, and S12. The Chao 1 estimator, that measures the “species” (in this case, OPFs) richness based on the distribution of “singletons” and “doubletons” (Chao, 1984), revealed that the richness of ARHD genes in the mangrove sediment was higher than in all other sites, except for site S9 (Amazonia, Table 2). Indeed, almost 82% of OPFs determined in this study were represented by a single clone.

**Table 2:** Diversity and richness indices of ARHD genes in the sites under study

Sites	Cutoff	OPFs	Clone numbers	ACE	Shannon	Simpson	Chao	Coverage (%)
SM	0.02	23	129	89.85	1.70	0.35	91.00	0.87
	0.05	11	129	0.00	0.82	0.62	47.00	0.93
S1	0.02	8	206	14.90	0.71	0.70	9.50	0.99
	0.05	4	206	4.00	0.64	0.70	4.00	1.00
S2	0.02	7	168	10.69	0.92	0.45	8.50	0.98
	0.05	1	168	0.00	0.00	1.00	1.00	1.00
S3	0.02	12	79	97.01	1.57	0.28	40.00	0.90
	0.05	6	79	13.60	0.94	0.48	9.00	0.96
S4	0.02	28	93	45.73	2.76	0.09	46.20	0.85
	0.05	15	93	16.74	2.15	0.16	15.75	0.97
S5	0.02	21	88	29.13	2.51	0.11	24.00	0.92
	0.05	19	88	29.87	2.42	0.12	21.50	0.93
S6	0.02	9	29	12.56	1.58	0.31	10.50	0.86
	0.05	6	29	6.46	1.35	0.33	6.00	0.97
S7	0.02	14	89	16.53	2.16	0.16	17.00	0.96
	0.05	8	89	8.45	1.74	0.21	8.00	0.99
S8	0.02	5	10	5.56	1.56	0.13	5.00	0.90
	0.05	3	10	3.00	1.03	0.31	3.00	1.00
S9	0.02	217	810	376.45	4.26	0.05	330.19	0.87
	0.05	78	810	103.21	3.07	0.11	99.43	0.97
S10	0.02	8	10	73.05	1.97	0.07	29.00	0.30
	0.05	7	10	16.44	1.83	0.09	12.00	0.50
S11	0.02	10	33	25.75	1.35	0.44	17.00	0.79
	0.05	6	33	6.50	1.14	0.45	6.00	0.97
S12	0.02	3	3	1.00	0.00	1.00	1.00	1.00
	0.05	3	3	1.00	0.00	1.00	1.00	1.00
S13	0.02	9	12	26.79	2.09	0.06	19.50	0.42
	0.05	7	12	17.45	1.75	0.14	17.00	0.58
S15	0.02	5	8	8.69	1.49	0.17	6.50	0.63
	0.05	5	8	8.69	1.49	0.14	6.50	0.63

**Figure 5:**
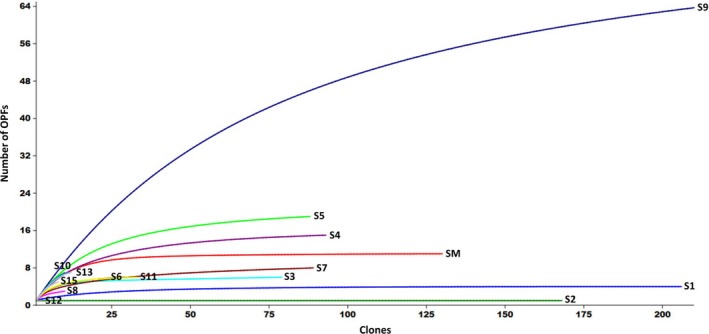
Rarefaction curves representing the richness of ARHDs (number of OPFs) as a function of the number of cloned gene sequences analyzed for each site. Information about the sites is detailed in Table 1

The rarefaction curve expressed the observed number (richness) of OPFs for each site as a function of the total sequences recovered from each site (Sanders, 1968). The complete coverage of dataset is expected to result in a plateau‐shaped curve. The results showed that the sampling effort performed in this study was sufficient to reveal all the OPFs present in the samples from sites SM (Mangrove sediment), S1 (China), S2 (Northeast Brazil), S3 (Maritime Antarctica) and S9 (Amazonia) (Figure 5). For the sites S4, S5, S6, S7 and S11, the curve clearly tends to an asymptote and additional sampling will probably reveal only a few number of additional OPFs. Finally, for the sites S8, S10, S12, S13, and S15, the weakly curvilinear plots showed that OPFs richness is far from being complete and further sampling may reveal potential untapped diversity (Figure 5).

**Figure 6:**
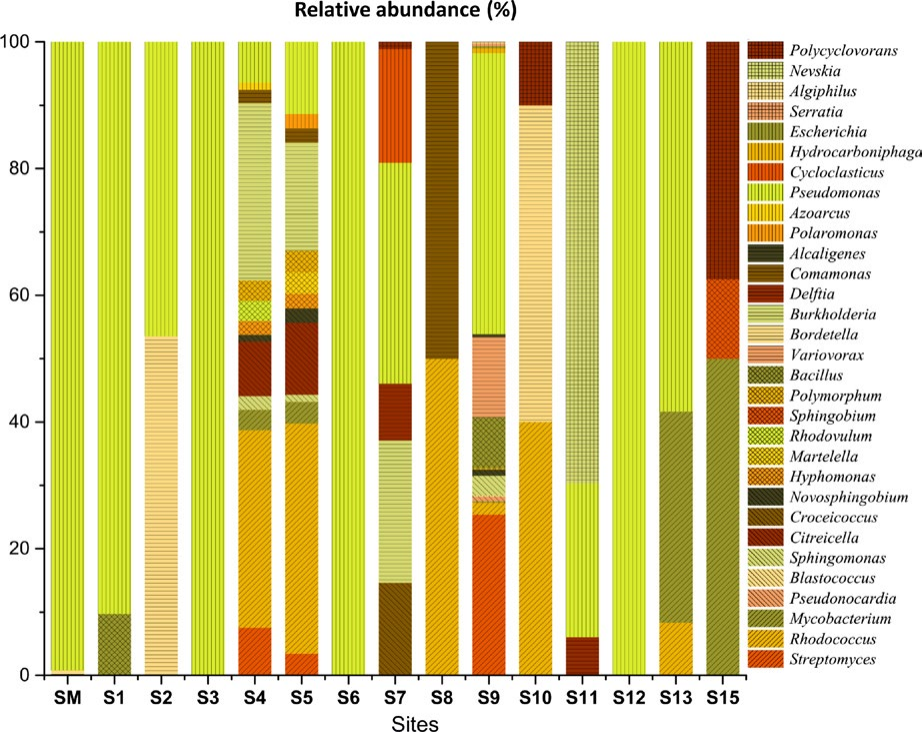
Distribution of genera in each site based on the relative abundance of ARHD sequences annotated against the NCBI RefSeq database by BLAST

The analysis of taxonomic diversity and abundance showed that *Pseudomonas* was the most common genus related to the ARHDs among the sites examined (Figure 6). The sites S4, S5, and S9 showed the highest richness of bacterial genera, suggesting that the higher diversity of dioxygenases observed in these sites (Table 2) is spread among distinct taxonomic groups. Curiously, there was not much taxonomic disparity between sites S4 and S5, which are geographically closely located. Nevertheless, detailed analysis of these sites revealed small differences in their taxonomic abundance. The *Rhodococcus*,* Citreicella*, and *Pseudomonas* genera were more abundant in the site S5 (Antarctica) than in the S4 (Antarctica), whereas *Burkholderia* and *Streptomyces* showed the opposite pattern. In addition, the genera *Martelella* and *Polaromonas* (S5), and *Rhodovulum* and *Azoarcus* (S4) appear only on these sites. The diversity of genera inferred from the dioxygenase sequences found in the mangrove sediment was low, although approximately half of the OPFs found presented average identity lower than 80% with their closest bacterial hits.

**Figure 7:**
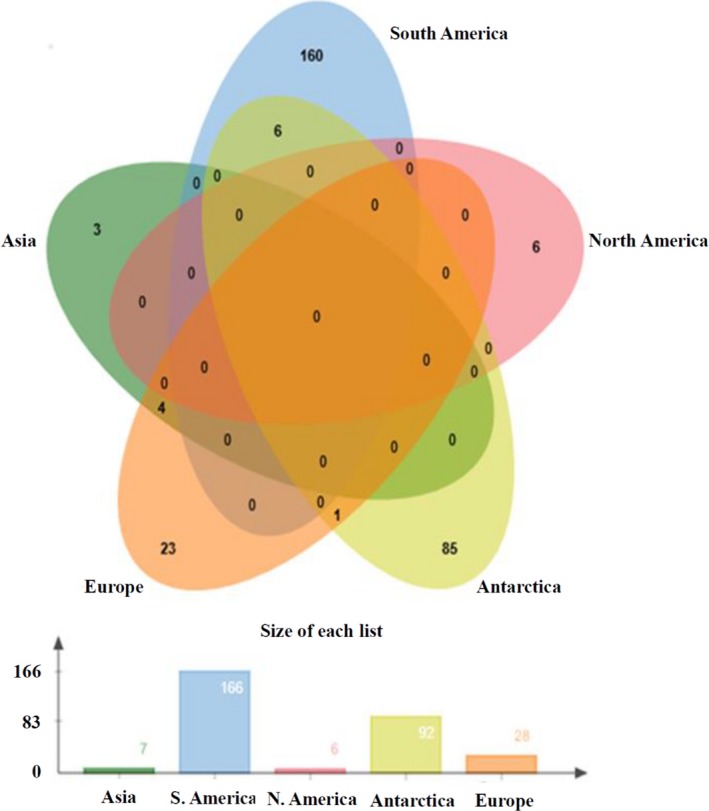
Venn diagram of shared OPFs among sites of different continental regions including Asia (site S1), North America (site S8), South America (sites S2, S7, and S9), Europe (sites S6, S10, and S13) and Antarctica (sites S3, S4, S5, S11, S12, and S15). The bar graph shows the number of OPFs found in each region. Detailed information on the sites is shown in Table 1. This analysis was run using the jvenn online platform (Bardou et al., 2014)

The Venn diagram showed the shared OPFs among sites of different continental regions. According to the data, 3, 160, 6, 85, and 23 OPFs showed endemism in Asia (site S1), South America (sites S2, S7, and S9), North America (site S8), Antarctica (sites S3, S4, S5, S11, S12, and S15) and Europe (sites S6, S10, and S13), respectively. In contrast, Europe shared one OPF with Antarctica and four OPFs with Asia, and South America shared six OPFs with Antarctica (Figure 7).

### 3.6 Principal coordinates analysis of the ARHD genes

**Figure 8:**
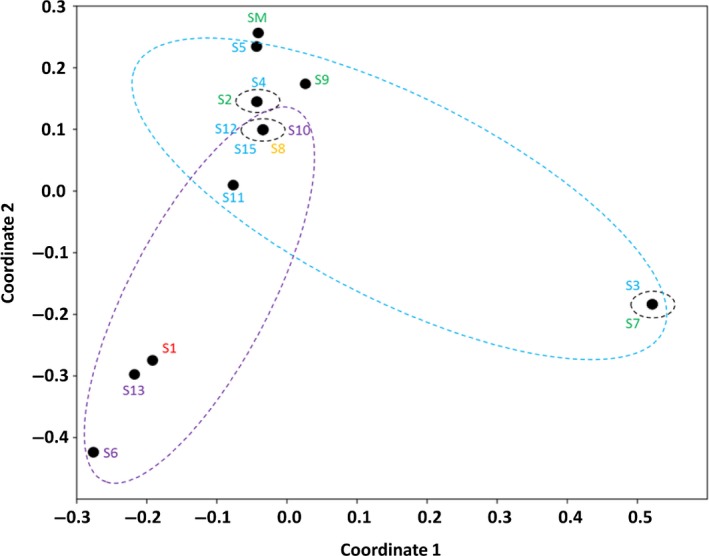
The Principal Coordinates Analysis (PCoA) based on Bray–Curtis distances showing the genetic correlation among the ARHD gene sequences worldwide. The clustering of the samples from Asia (red), North America (orange), South America (green), Europe (purple), and Antarctic (blue) are highlighted by the colored circles

Principal coordinates analysis (PCoA) of Bray–Curtis distances revealed significant correlations among sites as a function of shared OPFs. The majority of the sites were closely grouped respecting their continental origin. The sites of South America (SM, S9 e S2), Antarctica (S4, S5, S11, S12, and S15) and Europe (S6 and S13) grouped together. Although S3 and S7 sites are located in Antarctica and South America (Patagonia Coast), respectively, they showed larger discrepancy from the other sites with the same geographical location. The site S8, the only one located in North America, grouped together with the majority of the sites from South America and Antarctica. On the other hand, the site S1, the only one located in Asia, grouped together with the sites S6 and S13, both located in France (Figure 8).

## 4 DISCUSSION

In this study, the biogeographic distribution patterns of ARHD functional genes obtained from 15 different sites worldwide was investigated, in addition to their diversity at the functional and taxonomic levels.

According to the functional assignment of the OPFs, benzene 1,2‐dioxygenase, 3‐phenylpropionate dioxygenase, naphthalene 1,2‐dioxygenase, IPB‐dioxygenase, phenoxybenzoate dioxygenase and catechol 2,3‐dioxygenase were the most abundant enzyme sequences reported in this study, all involved in the aromatic hydrocarbon degradation. All these functions have already been grouped into dioxygenase families from the classification systems proposed previously (Batie, LaHaie, & Ballou, 1987; Gibson & Parales, 2000; Nam et al., 2001). Recently, the work of Iwai et al. (2011) has been suggested as the best study exploring the diversity and phylogenetic classification of dioxygenases derived from different environmental samples. According with their analysis, the dioxygenase genes were grouped into five subclades: PAH‐GN (PAH dioxygenases from Gram‐negative bacteria), PAH‐GP (PAH dioxygenases from Gram‐positive bacteria), T/B (toluene/biphenyl dioxygenases), OT‐I (other dioxygenases I) and OT‐II (other dioxygenases II). In addition, a large gene cluster was classified as benzoate dioxygenases or not functionally characterized Rieske‐type dioxygenases. More recently, Meynet, Head, Werner, and Davenport (2015) published a re‐evaluation of dioxygenase gene‐based phylogeny that showed, on one side, subclades with closely related genes derived from microorganisms that degrade the same substrate, and, on the other side, clades encompassing dioxygenase genes derived from microorganisms able to degrade different substrates. In the present study, lineages 1, 2, and 3 represented PAH‐GN, T/B and PAH‐GP dioxygenases, respectively, similarly grouped in previous articles (Gibson & Parales, 2000; Iwai et al., 2011), whereas the lineage 4 showed similarity with phenylpropionate/phthalate/aromatic dioxygenases and benzoate/aromatic dioxygenases. All enzymes showed correlation with the dioxygenase groups indicated by the studies cited above. Benzene 1,2‐dioxygenase and IPB‐dioxygenase were showed as the closest clades in the phylogenetic tree, as suggested by Gibson and Parales (2000) and Nam et al., 2001;. Naphthalene 1,2‐dioxygenase, phenoxybenzoate dioxygenase, 3‐phenylpropionate dioxygenase and catechol 2,3‐dioxygenase represented the groups III, I (Nam et al., 2001), clade C into lineage 2 and clade D into lineage 3 respectively, according to previous classifications (Meynet et al., 2015).

The present work revealed interesting patterns of ARHD biogeographic distribution. Integrated phylogenetic analysis of deduced peptide sequences from OPFs of each site examined revealed two aspects: (1) site‐specific genetic endemism for some of the ARHD families (e.g., some benzene 1,2‐ dioxygenases, IPB dioxygenases, phenoxybenzoate dioxygenases and some undefined α‐ARHD families), especially in Antarctic and Amazonian sites; and (2) genetic congruence among several ARHDs genes obtained from different environments, even separated by large distances. Müller et al. (2015) carried out an investigation about phylogenetic diversity and environmental distribution of *dsrAB* genes, coding for a reductase, and reported that members of the same *dsrAB* lineage were allocated in contrasting environments. On the other hand, the same work reported that there are unique lineages inhabiting environments with specific biogeochemical properties. In this study, some ARHD OPFs obtained from geographically different environments presented genetic relatedness. In some cases, such as the clustering between the sites SM (oil‐spilled mangrove sediments from Brazilian southeast coast) and S5 (sediments from Admiralty Bay, King George Island), or between the sites S3 (soil from Potter Peninsula & Jubany Station, King George Island) and S7 (coastal marine sediments from Patagonia), the evolutionary relatedness among OPFs might be explained by transport of microorganisms between these sites through ocean currents. In other cases, such as the clustering between the sites S8 (Polychlorinated Biphenyl‐Contaminated River Raisin Sediment, southeastern Michigan, United States) and S10 (Pine root zone contaminated with PCBs, Czech Republic), the relatedness among ARHDs OPFs could be explained by evolutionary convergence as a function of the substrate nature (PCBs). On the other hand, clustering of the sites S1 (sediments from the Pearl River estuary, China) and S13 (PAH‐polluted soil, Neuves‐Maisons, France), or of the sites S2 (oil samples from Potiguar Basin, Northeast Brazil) and S4 (sediments from Botany Point, King George island, Antarctica) showed no apparent correlation, suggesting that, in some cases, environmental factors do not define the evolution of genetic lineages. Finally, there were some OPFs that clustered together according to their biogeochemical origins, demonstrating ecological coherence (OPFs from sites S5, S4, S12, S15 and S11; and OPFs from sites S13 and S6). The concept of ecological coherence was well employed in the study of Oton et al., 2015, when they discovered a correlation between physicochemical properties and genetic diversity in soil samples.

*Pseudomonas*,* Streptomyces*,* Variovorax*,* Bordetella*, and *Rhodococcus* were the most abundant genera inferred from ARHD sequences recovered from all sites examined. However, the different protocols of DNA extraction, primers used, Polymerase Chain Reaction (PCR), number of sequences and other experimental procedures used may have limited the access to the taxonomic diversity in sites SM, S1, S2, S3, S6, S8 e S12. On the other hand, although the inferred taxonomic diversity has been low at some sites (SM, S1, S2, and S3), the richness of OPFs determined was high, as shown in the rarefaction curves (Figure 5). Despite different primer sets have been used across all the analyzed studies, all of them targeted the same conserved gene region for ARHDs.

Several species of the genus *Pseudomonas* have been extensively reported with potential for degradation of aromatic hydrocarbons (De Lima‐Morales et al., 2016). Metagenomic analysis carried out in the same site investigated in this study revealed the predominance of Gammaproteobacteria (Andreote et al., 2012), an important class of bacteria that encompasses *Pseudomonas,* known for their aromatic hydrocarbon degradation ability. Similarly, the ability of Actinobacteria members, such as *Streptomyces* and *Rhodococcus*, to metabolize hydrocarbons, including toluene, ethylbenzene, xylenes, biphenyl, polycyclic aromatic compounds and phenolic compounds, and their bioremediation potential has been well documented (Balachandran, Duraipandiyan, Balakrishna, & Ignacimuthu, 2012; Ishiyama, Vujaklija, & Davies, 2004; Martínková et al., 2009). The potential for aromatic compound degradation, based on functional and genetic evidences, is also well established for members of the Betaproteobacteria class, especially from the Burkholderiales order, such as the genera *Variovorax* and *Bordetella* (Satola, Wübbeler, & Steinbüchel, 2013). Thus, the data obtained in this study on the taxonomic affiliation of the ARHD sequences are strongly corroborated by previous findings.

The Venn diagram revealed that the South America and Antarctic sites shared microorganisms containing the same allele gene for dioxygenase in their genomes. Similar situation occurred between Asia and Europe, which shared four alleles. This finding suggests that some dispersal mechanisms (such as wind, marine currents or host organisms) may have allowed the transfer of gene content between these two regions. On the other hand, the endemism seen in the continental regions could be explained by their intrinsic environmental characteristics and/or by the long distance among them. The oceans can also play the role of geographic barrier, favoring the speciation process of microbial groups by mutations, isolation and reproduction. These long distances and geographic barriers could answer why there were no alleles shared between Asia and North America, Europe and North America, Europe and South America, Antarctica and Asia and Antarctica and North America.

The phylogenetic analysis of the ARHD OPFs was in accordance with the clustering of sites by PCoA. The grouping of the Antarctic samples could be explained by the influence of environmental factors, such as temperature and nutrient availability, which might have shaped the genetic diversity of the ARHDs in the microbial community. Previous studies have already showed that water depth, temperature, nutrient composition and salinity can shape the microbial community in nature (Cao, Hong, Li, & Gu, 2012; Oton et al., 2015). The close genetic relatedness among different environments (such as S4, S5, S11, S12, and S15) could be explained by microbial dispersal across ocean currents. According to Hanson et al. (2012), most studies show that microorganisms show limited dispersal due to their low mobility, favoring its establishment in restricted areas. On the other hand, the movement of microorganisms across space may occur by passive dispersal (e.g., via wind and air) or active dispersal (e.g., via ride in larger organisms), facilitating gene flow among different geographic regions (Martiny et al., 2006; Nemergut et al., 2013). Smith et al. (2013) revealed the dispersal of microorganisms across air masses and evidenced the presence of phyla with adaptations for atmospheric displacement. Microbial dispersion can also occur across ocean currents and thermohaline circulation (McGillicuddy et al., 2007). Hamdan et al. (2013) showed that hydrography can shape local and global microbial communities.

The phylogenetic tree showed some divergences in the distribution of the taxonomic classes inferred from the ARHD sequences. These divergences may be related to two factors: (1) low percentage of genetic similarity with their closest bacterial hits in the database; or (2) horizontal gene transfer. The lack of accuracy and representativeness of the available databases may be one of the causes for the first factor. The second factor is a natural biological phenomenon driven by selective pressures and may lead to taxonomic divergences. The study conducted by Jacob Parnell et al. (2010) showed that microbial biogeography may be affected by horizontal gene transfer, resulting in an average similarity of functional genes higher than the taxonomic average similarity evaluated based on the 16S ribosomal gene (Jacob Parnell et al., 2010). It is already known that horizontal gene transfer raises doubts about the veracity of the likely taxonomic classification inferred from the genetic elements that move among bacterial and archaeal species. In this study, a more robust analysis would be necessary to evaluate the percentage of phylogenetic congruence between 16S rRNA and ARHD genes from several bacterial genera. Oton et al. (2015) reported 85% of phylogenetic congruence between 16S rRNA and *amoA* genes of Thaumarchaeota, demonstrating low horizontal transfer rate of these genes.

In conclusion, this study contributed to the knowledge on the richness, distribution, and genetic relatedness of the ARHD genes recovered from fifteen different sites, as well as the taxonomic groups likely associated with such functional genes. A moderate genetic congruence was evidenced among ARHDs worldwide and, in some cases, biogeographic endemism patterns were revealed, presumably modeled by biotic and abiotic environmental factors. Results suggest that there was a connectivity between South America and Antarctica sites, probably caused by sea currents, air masses or host organisms. This could have facilitated the dispersion of microorganisms between these two regions, helping to explain their evolutionary history.

## CONFLICTS OF INTEREST

None.
