# Supplementary material for: Diversity of aromatic hydroxylating dioxygenase genes in mangrove microbiome and their biogeographic patterns across global sites
Source: Microbiologyopen. 2017 May 23;6(4):e00490. doi: 10.1002/mbo3.490 (PMC5552929; doi:10.1002/mbo3.490)
Supplement: Supplementary file 1 [file MBO3-6-na-s001.docx]

**Table S1**: Functional and taxonomic annotation of all OPFs.

| **OPF IDs** | **Abundance** | **Enzyme family** | **Closest bacterial hit** | **Ident** | **Accession** |
| --- | --- | --- | --- | --- | --- |
| >SM_OPF001 | 101 | benzene 1,2-dioxygenase subunit alpha | Pseudomonas | 97% | WP_012052601.1 |
| >SM_OPF002 | 20 | benzene 1,2-dioxygenase subunit alpha | Pseudomonas | 92% | WP_012052601.1 |
| >SM_OPF003 | 1 | benzene 1,2-dioxygenase subunit alpha | Pseudomonas | 74% | WP_012052601.1 |
| >SM_OPF004 | 1 | benzene 1,2-dioxygenase subunit alpha | Pseudomonas | 68% | WP_012052601.1 |
| >SM_OPF005 | 1 | benzene 1,2-dioxygenase subunit alpha | Pseudomonas | 82% | WP_012052601.1 |
| >SM_OPF006 | 1 | benzene 1,2-dioxygenase subunit alpha | Pseudomonas | 94% | WP_012052601.1 |
| >SM_OPF007 | 1 | benzene 1,2-dioxygenase subunit alpha | Pseudomonas | 89% | WP_012052601.1 |
| >SM_OPF008 | 1 | benzene 1,2-dioxygenase | Bordetella petrii | 70% | WP_041863076.1 |
| >SM_OPF009 | 1 | benzene 1,2-dioxygenase subunit alpha | Pseudomonas | 68% | WP_012052601.1 |
| >SM_OPF010 | 1 | benzene 1,2-dioxygenase subunit alpha | Pseudomonas | 45% | WP_012052601.1 |
| >SM_OPF011 | 1 | benzene 1,2-dioxygenase subunit alpha | Pseudomonas | 64% | WP_012052601.1 |
| >S1_OPF001 | 171 | naphthalene dioxygenase Fe-S protein large subunit | Pseudomonas putida | 91% | WP_011154413.1 |
| >S1_OPF002 | 15 | naphthalene dioxygenase Fe-S protein large subunit | Pseudomonas putida | 98% | WP_011154413.1 |
| >S1_OPF003 | 10 | 3-phenylpropionate dioxygenase | Bacillus massiliosenegalensis | 58% | WP_019153061.1 |
| >S1_OPF004 | 10 | 3-phenylpropionate dioxygenase | Bacillus aidingensis | 52% | WP_026702167.1 |
| >S2_OPF001 | 90 | benzene 1,2-dioxygenase | Bordetella petrii | 88% | WP_041863076.1 |
| >S2_OPF002 | 78 | benzene 1,2-dioxygenase | Pseudomonas frederiksbergensis | 93% | WP_039594666.1 |
| >S3_OPF01 | 50 | naphthalene dioxygenase Fe-S protein large subunit | Pseudomonas putida | 100% | WP_011154413.1 |
| >S3_OPF02 | 23 | naphthalene dioxygenase iron sulfur protein large subunit | Pseudomonas | 96% | WP_011475377.1 |
| >S3_OPF03 | 3 | naphthalene dioxygenase Fe-S protein large subunit | Pseudomonas putida | 90% | WP_011154413.1 |
| >S3_OPF04 | 1 | naphthalene 1,2-dioxygenase subunit alpha | Pseudomonas | 96% | WP_011117400.1 |
| >S3_OPF05 | 1 | naphthalene dioxygenase Fe-S protein large subunit | Pseudomonas putida | 86% | WP_011154413.1 |
| >S3_OPF06 | 1 | naphthalene dioxygenase Fe-S protein large subunit | Pseudomonas putida | 98% | WP_011154413.1 |
| >S4_OPF01 | 26 | biphenyl dioxygenase subunit alpha | Burkholderia xenovorans | 100% | WP_011494299.1 |
| >S4_OPF02 | 24 | IPB-dioxygenase ISP large subunit (IpbA1) | Rhodococcus erythropolis | 100% | WP_011133490.1 |
| >S4_OPF03 | 8 | benzene 1,2-dioxygenase | Citreicella sp. 357 | 99% | WP_009505788.1 |
| >S4_OPF04 | 6 | 3-phenylpropionate dioxygenase | Streptomyces ossamyceticus | 54% | WP_055520670.1 |
| >S4_OPF05 | 6 | benzene 1,2-dioxygenase subunit alpha | Pseudomonas | 100% | WP_012052601.1 |
| >S4_OPF06 | 5 | IPB-dioxygenase ISP large subunit (IpbA1) | Rhodococcus erythropolis | 91% | WP_011133490.1 |
| >S4_OPF07 | 3 | benzene 1,2-dioxygenase | Rhodovulum sp. NI22 | 56% | WP_037209959.1 |
| >S4_OPF08 | 3 | 3-phenylpropionate dioxygenase | Mycobacterium | 81% | WP_011781569.1 |
| >S4_OPF09 | 3 | benzene 1,2-dioxygenase | Polymorphum gilvum | 91% | WP_041375675.1 |
| >S4_OPF10 | 2 | benzene 1,2-dioxygenase | Comamonas testosteroni | 99% | WP_043371571.1 |
| >S4_OPF11 | 2 | benzene 1,2-dioxygenase | Sphingomonas sp. Ant20 | 64% | WP_037529249.1 |
| >S4_OPF12 | 2 | benzene 1,2-dioxygenase | Hyphomonas chukchiensis | 57% | WP_051615035.1 |
| >S4_OPF13 | 1 | 3-phenylpropionate dioxygenase | Streptomyces ossamyceticus | 55% | WP_055520670.1 |
| >S4_OPF14 | 1 | 3-phenylpropionate dioxygenase | Novosphingobium nitrogenifigens | 53% | WP_008070358.1 |
| >S4_OPF15 | 1 | benzene 1,2-dioxygenase | Azoarcus sp. CIB | 91% | WP_050417871.1 |
| >S5_OPF01 | 22 | IPB-dioxygenase ISP large subunit (IpbA1) | Rhodococcus erythropolis | 100% | WP_011133490.1 |
| >S5_OPF02 | 14 | biphenyl dioxygenase subunit alpha | Burkholderia xenovorans | 100% | WP_011494299.1 |
| >S5_OPF03 | 10 | benzene 1,2-dioxygenase | Citreicella sp. 357 | 99% | WP_009505788.1 |
| >S5_OPF04 | 9 | IPB-dioxygenase ISP large subunit (IpbA1) | Rhodococcus erythropolis | 91% | WP_011133490.1 |
| >S5_OPF05 | 8 | benzene 1,2-dioxygenase subunit alpha | Pseudomonas | 100% | WP_012052601.1 |
| >S5_OPF06 | 3 | benzene 1,2-dioxygenase | Martelella sp. AD-3 | 56% | WP_036236102.1 |
| **OPF IDs** | **Abundance** | **Enzyme family** | **Closest bacterial hit** | **Ident** | **Accession** |
| >S5_OPF07 | 3 | 3-phenylpropionate dioxygenase | Mycobacterium | 80% | WP_011781569.1 |
| >S5_OPF08 | 3 | 3-phenylpropionate dioxygenase | Streptomyces ossamyceticus | 54% | WP_055520670.1 |
| >S5_OPF09 | 2 | benzene 1,2-dioxygenase | Comamonas testosteroni | 99% | WP_043371571.1 |
| >S5_OPF10 | 2 | benzene 1,2-dioxygenase | Polymorphum gilvum | 91% | WP_041375675.1 |
| >S5_OPF11 | 2 | 3-phenylpropionate dioxygenase | Novosphingobium nitrogenifigens | 53% | WP_008070358.1 |
| >S5_OPF12 | 2 | benzene 1,2-dioxygenase | Polaromonas naphthalenivorans | 94% | WP_011797818.1 |
| >S5_OPF13 | 2 | benzene 1,2-dioxygenase | Hyphomonas chukchiensis | 57% | WP_051615035.1 |
| >S5_OPF14 | 1 | benzene 1,2-dioxygenase | Pseudomonas toyotomiensis | 81% | WP_059391947.1 |
| >S5_OPF15 | 1 | IPB-dioxygenase ISP large subunit (IpbA1) | Rhodococcus erythropolis | 85% | WP_011133490.1 |
| >S5_OPF16 | 1 | benzene 1,2-dioxygenase | Polymorphum gilvum | 95% | WP_041375675.1 |
| >S5_OPF17 | 1 | biphenyl dioxygenase subunit alpha | Burkholderia xenovorans | 98% | WP_011494299.1 |
| >S5_OPF18 | 1 | benzene 1,2-dioxygenase | Sphingomonas sp. Ant20 | 64% | WP_037529249.1 |
| >S5_OPF19 | 1 | benzene 1,2-dioxygenase | Pseudomonas aeruginosa | 99% | WP_059400052.1 |
| >S6_OPF01 | 16 | naphthalene 1,2-dioxygenase subunit alpha | Pseudomonas | 100% | WP_011117400.1 |
| >S6_OPF02 | 5 | naphthalene 1,2-dioxygenase subunit alpha | Pseudomonas | 91% | WP_011117400.1 |
| >S6_OPF03 | 3 | naphthalene 1,2-dioxygenase subunit alpha | Pseudomonas | 95% | WP_011117400.1 |
| >S6_OPF04 | 2 | naphthalene 1,2-dioxygenase subunit alpha | Pseudomonas | 94% | WP_011117400.1 |
| >S6_OPF05 | 2 | naphthalene 1,2-dioxygenase subunit alpha | Pseudomonas | 91% | WP_011117400.1 |
| >S6_OPF06 | 1 | naphthalene dioxygenase iron sulfur protein large subunit | Pseudomonas | 90% | WP_011475377.1 |
| >S7_OPF01 | 31 | naphthalene dioxygenase Fe-S protein large subunit | Pseudomonas putida | 100% | WP_011154413.1 |
| >S7_OPF02 | 20 | naphthalene 1,2-dioxygenase | Burkholderia sp. Ch1-1 | 99% | WP_007179244.1 |
| >S7_OPF03 | 13 | naphthalene 1,2-dioxygenase | Croceicoccus naphthovorans | 60% | WP_047820944.1 |
| >S7_OPF04 | 8 | naphthalene 1,2-dioxygenase | Delftia sp. Cs1-4 | 69% | WP_013801305.1 |
| >S7_OPF05 | 7 | PAH dioxygenase iron sulfur protein large subunit | Cycloclasticus | 100% | WP_016391028.1 |
| >S7_OPF06 | 7 | naphthalene 1,2-dioxygenase | Cycloclasticus | 61% | WP_015007028.1 |
| >S7_OPF07 | 2 | naphthalene 1,2-dioxygenase | Cycloclasticus | 61% | WP_015005786.1 |
| >S7_OPF08 | 1 | naphthalene 1,2-dioxygenase | Polycyclovorans algicola | 96% | WP_029889175.1 |
| >S8_OPF01 | 5 | benzene 1,2-dioxygenase | Comamonas testosteroni | 92% | WP_057092204.1 |
| >S8_OPF02 | 3 | benzene 1,2-dioxygenase | Rhodococcus erythropolis | 82% | WP_042445407.1 |
| >S8_OPF03 | 2 | benzene 1,2-dioxygenase | Rhodococcus opacus | 76% | WP_012687209.1 |
| >S9_OPF001 | 48 | benzene 1,2-dioxygenase | Bacillus | 93% | WP_048680352.1 |
| >S9_OPF002 | 73 | aromatic-ring-hydroxylating dioxygenase subunit alpha | Variovorax paradoxus | 56% | WP_021006872.1 |
| >S9_OPF003 | 39 | 3-phenylpropionate dioxygenase | Streptomyces ossamyceticus | 62% | WP_055520670.1 |
| >S9_OPF004 | 34 | 3-phenylpropionate dioxygenase | Streptomyces ossamyceticus | 64% | WP_055520670.1 |
| >S9_OPF005 | 3 | biphenyl 2,3-dioxygenase subunit alpha | Rhodococcus rhodnii | 58% | WP_010839881.1 |
| >S9_OPF006 | 13 | 3-phenylpropionate dioxygenase | Streptomyces ossamyceticus | 94% | WP_055520670.1 |
| >S9_OPF007 | 10 | 3-phenylpropionate dioxygenase | Bacillus massiliogorillae | 55% | WP_042349570.1 |
| >S9_OPF008 | 10 | 3-phenylpropionate dioxygenase | Streptomyces ossamyceticus | 63% | WP_055520670.1 |
| >S9_OPF009 | 8 | 3-phenylpropionate dioxygenase | Streptomyces ossamyceticus | 59% | WP_055520670.1 |
| >S9_OPF010 | 297 | benzene 1,2-dioxygenase subunit alpha | Pseudomonas | 95% | WP_012052601.1 |
| >S9_OPF011 | 47 | 3-phenylpropionate dioxygenase | Streptomyces ossamyceticus | 62% | WP_055520670.1 |
| >S9_OPF012 | 2 | 3-phenylpropionate dioxygenase | Streptomyces zinciresistens | 72% | WP_007499173.1 |
| >S9_OPF013 | 2 | benzene 1,2-dioxygenase | Polymorphum gilvum | 48% | WP_041375675.1 |
| >S9_OPF014 | 2 | 3-phenylpropionate dioxygenase | Streptomyces ossamyceticus | 61% | WP_055520670.1 |
| >S9_OPF015 | 3 | 3-phenylpropionate dioxygenase | Streptomyces ossamyceticus | 59% | WP_055520670.1 |
| **OPF IDs** | **Abundance** | **Enzyme family** | **Closest bacterial hit** | **Ident** | **Accession** |
| >S9_OPF016 | 2 | benzene 1,2-dioxygenase | Bacillus | 90% | WP_048680352.1 |
| >S9_OPF017 | 4 | aromatic-ring-hydroxylating dioxygenase subunit alpha | Variovorax paradoxus | 57% | WP_021006872.1 |
| >S9_OPF018 | 5 | phenoxybenzoate dioxygenase | Streptomyces torulosus | 62% | WP_055713550.1 |
| >S9_OPF019 | 11 | aromatic-ring-hydroxylating dioxygenase subunit alpha | Variovorax paradoxus | 58% | WP_021006872.1 |
| >S9_OPF020 | 48 | naphthalene dioxygenase iron sulfur protein large subunit | Pseudomonas | 97% | WP_011475377.1 |
| >S9_OPF021 | 3 | 3-phenylpropionate dioxygenase | Novosphingobium nitrogenifigens | 61% | WP_008070358.1 |
| >S9_OPF022 | 2 | aromatic-ring-hydroxylating dioxygenase subunit alpha | Variovorax paradoxus | 58% | WP_021006872.1 |
| >S9_OPF023 | 2 | aromatic-ring-hydroxylating dioxygenase subunit alpha | Variovorax paradoxus | 58% | WP_021006872.1 |
| >S9_OPF024 | 2 | naphthalene 1,2-dioxygenase | Algiphilus aromaticivorans | 56% | WP_052367460.1 |
| >S9_OPF025 | 2 | biphenyl 2,3-dioxygenase subunit alpha | Rhodococcus rhodnii | 56% | WP_010839881.1 |
| >S9_OPF026 | 2 | 3-phenylpropionate dioxygenase | Streptomyces ossamyceticus | 60% | WP_055520670.1 |
| >S9_OPF027 | 1 | phenylpropionate dioxygenase | Streptomyces acidiscabies | 63% | WP_010355984.1 |
| >S9_OPF028 | 25 | benzene 1,2-dioxygenase | Sphingomonas sp. Ant20 | 47% | WP_037529249.1 |
| >S9_OPF029 | 1 | 3-phenylpropionate dioxygenase | Novosphingobium nitrogenifigens | 59% | WP_008070358.1 |
| >S9_OPF030 | 2 | benzene 1,2-dioxygenase | Bacillus sp. UNC41MFS5 | 95% | WP_026564730.1 |
| >S9_OPF031 | 3 | 3-phenylpropionate dioxygenase | Escherichia coli | 51% | WP_000211164.1 |
| >S9_OPF032 | 3 | aromatic-ring-hydroxylating dioxygenase subunit alpha | Variovorax paradoxus | 56% | WP_021006872.1 |
| >S9_OPF033 | 10 | phenylpropionate dioxygenase | Streptomyces acidiscabies | 60% | WP_010355984.1 |
| >S9_OPF034 | 1 | 3-phenylpropionate dioxygenase | Streptomyces ossamyceticus | 62% | WP_055520670.1 |
| >S9_OPF035 | 3 | 3-phenylpropionate dioxygenase | Streptomyces zinciresistens | 75% | WP_007499173.1 |
| >S9_OPF036 | 4 | IPB-dioxygenase ISP large subunit (IpbA1) | Rhodococcus erythropolis | 85% | WP_011133490.1 |
| >S9_OPF037 | 2 | aromatic-ring-hydroxylating dioxygenase subunit alpha | Variovorax paradoxus | 56% | WP_021006872.1 |
| >S9_OPF038 | 6 | (2Fe-2S) ferredoxin | Pseudonocardia acaciae | 62% | WP_028923111.1 |
| >S9_OPF039 | 4 | 3-phenylpropionate dioxygenase | Alcaligenes faecalis | 47% | WP_060186372.1 |
| >S9_OPF040 | 1 | 3-phenylpropionate dioxygenase | Novosphingobium nitrogenifigens | 60% | WP_008070358.1 |
| >S9_OPF041 | 1 | 3-phenylpropionate dioxygenase | Streptomyces ossamyceticus | 60% | WP_055520670.1 |
| >S9_OPF042 | 1 | benzene 1,2-dioxygenase subunit alpha | Pseudomonas | 98% | WP_012052601.1 |
| >S9_OPF043 | 1 | 3-phenylpropionate dioxygenase | Streptomyces zinciresistens | 57% | WP_007499173.1 |
| >S9_OPF044 | 2 | 3-phenylpropionate dioxygenase | Novosphingobium nitrogenifigens | 67% | WP_008070358.1 |
| >S9_OPF045 | 1 | 3-phenylpropionate dioxygenase | Mycobacterium | 67% | WP_011781569.1 |
| >S9_OPF046 | 1 | Large subunit naph/bph dioxygenase | Rhodococcus sp. EsD8 | 70% | WP_006933068.1 |
| >S9_OPF047 | 1 | phenylpropionate dioxygenase | Streptomyces acidiscabies | 62% | WP_010355984.1 |
| >S9_OPF048 | 1 | Large subunit naph/bph dioxygenase | Rhodococcus sp. EsD8 | 77% | WP_006933068.1 |
| >S9_OPF049 | 1 | ring-hydroxylating dioxygenase | Enterobacteriaceae bacterium strain FGI 57 | 52% | WP_015963414.1 |
| >S9_OPF050 | 1 | (2Fe-2S)-binding protein | Blastococcus saxobsidens | 45% | WP_014378242.1 |
| >S9_OPF051 | 7 | naphthalene dioxygenase Fe-S protein large subunit | Pseudomonas putida | 97% | WP_011154413.1 |
| >S9_OPF052 | 6 | 2Fe-2S ferredoxin | Streptomyces scabiei | 51% | WP_059078277.1 |
| >S9_OPF053 | 6 | naphthalene 1,2-dioxygenase | Hydrocarboniphaga effusa | 55% | WP_007184423.1 |
| >S9_OPF054 | 2 | 3-phenylpropionate dioxygenase | Streptomyces zinciresistens | 70% | WP_007499173.1 |
| >S9_OPF055 | 3 | 3-phenylpropionate dioxygenase | Serratia fonticola | 54% | WP_024530775.1 |
| >S9_OPF056 | 3 | phenoxybenzoate dioxygenase | Streptomyces torulosus | 63% | WP_055713550.1 |
| >S9_OPF057 | 2 | aromatic-ring-hydroxylating dioxygenase subunit alpha | Variovorax paradoxus | 57% | WP_021006872.1 |
| >S9_OPF058 | 2 | 3-phenylpropionate dioxygenase | Streptomyces ossamyceticus | 57% | WP_055520670.1 |
| >S9_OPF059 | 2 | 3-phenylpropionate dioxygenase | Streptomyces ossamyceticus | 59% | WP_055520670.1 |
| >S9_OPF060 | 2 | benzene 1,2-dioxygenase | Bacillus | 89% | WP_048680352.1 |
| **OPF IDs** | **Abundance** | **Enzyme family** | **Closest bacterial hit** | **Ident** | **Accession** |
| >S9_OPF061 | 2 | phenoxybenzoate dioxygenase | Streptomyces torulosus | 62% | WP_055713550.1 |
| >S9_OPF062 | 2 | phenoxybenzoate dioxygenase | Rhodococcus rhodnii | 83% | WP_051110981.1 |
| >S9_OPF063 | 1 | benzene 1,2-dioxygenase | Pseudomonas toyotomiensis | 95% | WP_059391947.1 |
| >S9_OPF064 | 1 | phenoxybenzoate dioxygenase | Rhodococcus rhodnii | 67% | WP_051110981.1 |
| >S9_OPF065 | 1 | benzene 1,2-dioxygenase subunit alpha | Pseudomonas | 92% | WP_012052601.1 |
| >S9_OPF066 | 1 | 3-phenylpropionate dioxygenase | Streptomyces ossamyceticus | 89% | WP_055520670.1 |
| >S9_OPF067 | 1 | 3-phenylpropionate dioxygenase | Streptomyces ossamyceticus | 60% | WP_055520670.1 |
| >S9_OPF068 | 1 | 3-phenylpropionate dioxygenase | Novosphingobium nitrogenifigens | 60% | WP_008070358.1 |
| >S9_OPF069 | 1 | phenoxybenzoate dioxygenase | Mycobacterium abscessus | 83% | WP_052617997.1 |
| >S9_OPF070 | 1 | 3-phenylpropionate dioxygenase | Streptomyces ossamyceticus | 86% | WP_055520670.1 |
| >S9_OPF071 | 1 | aromatic-ring-hydroxylating dioxygenase subunit alpha | Variovorax paradoxus | 57% | WP_021006872.1 |
| >S9_OPF071 | 1 | aromatic-ring-hydroxylating dioxygenase subunit alpha | Variovorax paradoxus | 53% | WP_021006872.1 |
| >S10_OPF01 | 3 | benzene 1,2-dioxygenase | Rhodococcus opacus | 99% | WP_012687209.1 |
| >S10_OPF02 | 2 | benzene 1,2-dioxygenase | Bordetella petrii | 58% | WP_041863076.1 |
| >S10_OPF03 | 1 | benzene 1,2-dioxygenase | Polycyclovorans algicola | 86% | WP_029889979.1 |
| >S10_OPF04 | 1 | benzene 1,2-dioxygenase | Bordetella petrii | 61% | WP_041863076.1 |
| >S10_OPF05 | 1 | benzene 1,2-dioxygenase | Bordetella petrii | 57% | WP_041863076.1 |
| >S10_OPF06 | 1 | benzene 1,2-dioxygenase | Rhodococcus erythropolis | 96% | WP_042445407.1 |
| >S10_OPF07 | 1 | benzene 1,2-dioxygenase | Bordetella petrii | 57% | WP_041863076.1 |
| >S11_OPF01 | 22 | naphthalene 1,2-dioxygenase | Nevskia ramosa | 92% | WP_022978279.1 |
| >S11_OPF02 | 4 | naphthalene 1,2-dioxygenase subunit alpha | Pseudomonas | 99% | WP_011117400.1 |
| >S11_OPF03 | 2 | naphthalene 1,2-dioxygenase subunit alpha | Pseudomonas | 87% | WP_011117400.1 |
| >S11_OPF04 | 2 | naphthalene 1,2-dioxygenase | Delftia sp. Cs1-4 | 96% | WP_013801305.1 |
| >S11_OPF04 | 2 | naphthalene 1,2-dioxygenase subunit alpha | Pseudomonas | 93% | WP_011117400.1 |
| >S11_OPF06 | 1 | naphthalene 1,2-dioxygenase | Nevskia ramosa | 88% | WP_022978279.1 |
| >S12_OPF1 | 3 | naphthalene 1,2-dioxygenase subunit alpha | Pseudomonas | 89% | WP_011117400.1 |
| >S13_OPF01 | 4 | naphthalene 1,2-dioxygenase subunit alpha | Pseudomonas | 97% | WP_011117400.1 |
| >S13_OPF02 | 3 | ribosomal subunit interface protein | Mycobacterium gilvum | 100% | WP_013470369.1 |
| >S13_OPF03 | 1 | ribosomal subunit interface protein | Mycobacterium rhodesiae | 99% | WP_014211473.1 |
| >S13_OPF04 | 1 | ribosomal subunit interface protein | Rhodococcus | 85% | WP_017681826.1 |
| >S13_OPF05 | 1 | naphthalene 1,2-dioxygenase subunit alpha | Pseudomonas | 90% | WP_011117400.1 |
| >S13_OPF06 | 1 | naphthalene 1,2-dioxygenase subunit alpha | Pseudomonas | 98% | WP_011117400.1 |
| >S13_OPF07 | 1 | naphthalene 1,2-dioxygenase subunit alpha | Pseudomonas | 87% | WP_011117400.1 |
| >S15_OPF1 | 3 | naphthalene 1,2-dioxygenase | Polycyclovorans algicola | 45% | WP_029889175.1 |
| >S15_OPF2 | 2 | ribosomal subunit interface protein | Mycobacterium gilvum | 96% | WP_013470369.1 |
| >S15_OPF3 | 1 | ribosomal subunit interface protein | Mycobacterium | 100% | WP_011559048.1 |
| >S15_OPF4 | 1 | ribosomal subunit interface protein | Mycobacterium gilvum | 100% | WP_011891532.1 |
| >S15_OPF5 | 1 | catechol 2,3-dioxygenase | Sphingobium yanoikuyae | 100% | WP_037508695.1 |
